# Amyand’s Hernia Presenting With Acute Enlargement of a Chronic Right Inguinal Hernia: A Case Report

**DOI:** 10.1155/cris/3262891

**Published:** 2026-09-08

**Authors:** Allen Barbarovich, Christopher Staley, Christopher Soika

**Affiliations:** ^1^ Northeast Georgia Medical Center, 743 Spring Street NE, Gainesville 30501, Georgia, USA, nghs.com

**Keywords:** appendectomy, appendicitis, Aymand’s hernia, hernia repair, inguinal hernia

## Abstract

Amyand’s hernia is a rare condition characterized by the presence of the vermiform appendix within an inguinal hernia sac, which may or may not be inflamed. We present the case of a 43‐year‐old male with a 3‐year history of right inguinal hernia who presented to the emergency department with acute enlargement of the hernia sac and associated discomfort. A contrast‐enhanced computed tomography scan of the abdomen and pelvis revealed a right inguinal hernia containing an inflamed appendix, consistent with acute appendicitis, with periappendiceal stranding but no abscess formation. The patient denied recent trauma or bowel habit changes, though he reported chronic constipation. This report highlights the importance of imaging in diagnosing rare hernia contents and discusses the management controversies associated with Amyand’s hernia.

## 1. Introduction

Amyand’s hernia, first described by Claudius Amyand in 1735, involves the presence of the appendix within an inguinal hernia sac, accounting for ~1% of all inguinal hernias and only 0.1% when complicated by appendicitis [1–3]. Due to its rarity and nonspecific symptoms, preoperative diagnosis remains challenging and often relies on imaging modalities such as CT. The treatment approach remains controversial and depends on factors such as the condition of the appendix, the presence of inflammation, and the patient’s overall status [4, 5]. Ultimately, a care team must consider the risk of immediate complication against the future risk; if the appendix is normal, excision prevents future appendicitis but now contaminates the surgical field, increasing the risk of infection and making the use of mesh for the repair untenable. If the appendix is abnormal, however, the above risks are even further exacerbated, increasing the likelihood of reherniation and disseminated infection. Deferring appendectomy, however, in both cases leaves the risk of future appendicitis, the latter risking worsening of clinical course and perforation with complex return to the operating theater [6].

## 2. Case Presentation

A 43‐year‐old male presented with progressive enlargement of a known right‐sided inguinal hernia, first noted 3 years prior and previously asymptomatic. Three days before presentation, he developed discomfort and marked scrotal swelling, approximately doubling in size. He denied trauma, exertion, or changes in activity. His history was notable for chronic constipation with bowel movements every 3 days without recent changes, vomiting, or gastrointestinal bleeding.

**Figure 1:**
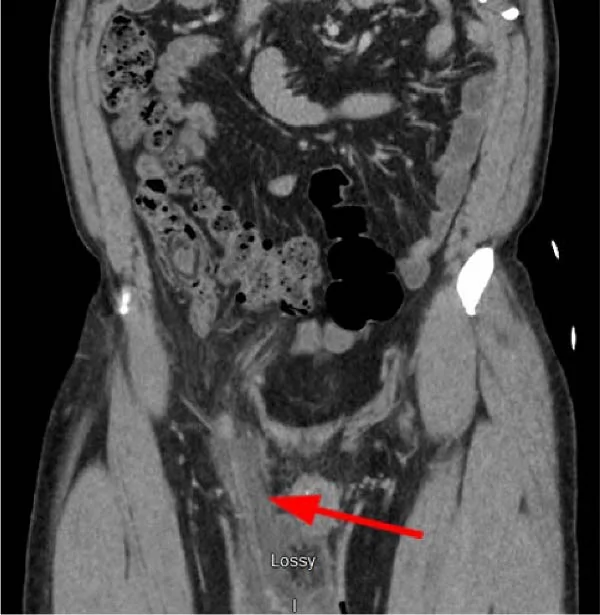
CT abdomen/pelvis. There is a fat‐containing hernia of the right inguinal region.

On examination, he was comfortable and hemodynamically stable. The right inguinal hernia was enlarged but non‐tender and reducible, without overlying skin changes or signs of peritonitis. Laboratory studies were unremarkable. CT abdomen and pelvis with contrast demonstrated a right inguinal hernia containing an inflamed appendix with periappendiceal fat stranding and fluid, consistent with acute appendicitis within the hernia sac (Figure 1). No abscess was identified. An incidental fat‐containing umbilical hernia was also noted.

**Figure 2:**
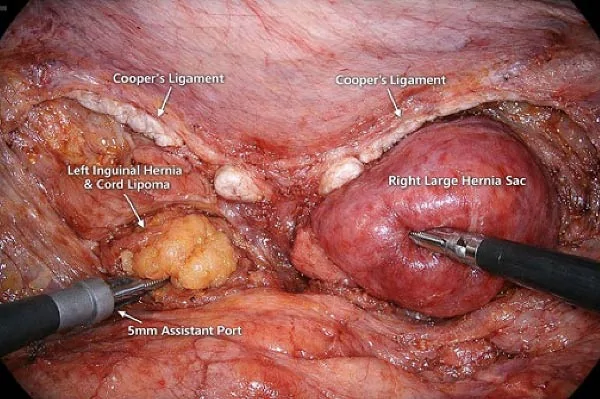
Anatomical view of the dissection plane described, with Cooper’s ligament and pubic tubercles as anatomical landmarks for subsequent reduction and mesh placement.

The patient underwent robotic‐assisted laparoscopic bilateral inguinal hernia repair with concomitant appendectomy. Intraoperative findings demonstrated bilateral indirect inguinal hernias, with the right‐sided hernia containing the inflamed appendix, consistent with Amyand’s hernia. Intraoperatively, a large amount of omentum was reduced from the right hernia. The appendix was found to be tethered within the hernia sac and not fully reducible. A peritoneal flap was created and extended bilaterally to expose the inguinal regions. The left inguinal hernia was reduced without difficulty, while the right‐sided hernia required additional traction and partial transection of the sac due to its large size (Figure 2).

**Figure 3:**
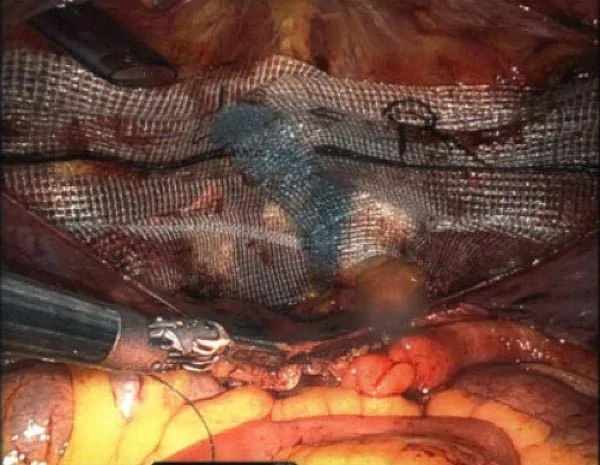
Intraoperative image of mesh positioning in the preperitoneal dissection plane.

**Figure 4:**
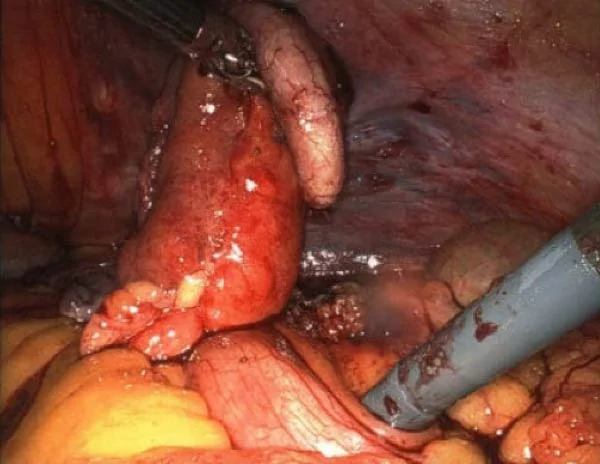
Intraoperative image of the edematous appendix isolated subsequent to hernia repair and closure, with plastered hernia sac visible.

Following reduction, a bilateral ProGrip mesh was placed, with appropriate overlap at the midline (Figure 3). The peritoneum was closed over the mesh. The appendix was identified, ligated at its base, divided, and removed, with careful hemostasis of the mesoappendix (Figure 4).

The patient tolerated the procedure well. At the 2‐week follow‐up, he demonstrated mild scrotal seroma without other complications, requiring no further intervention.

## 3. Discussion

Amyand’s hernia remains a diagnostic and therapeutic challenge due to its rare presentation and overlap with more common inguinal hernia symptoms [2, 7]. Most cases are identified intraoperatively; however, the increasing use of cross‐sectional imaging, particularly CT, has improved preoperative identification [8–10]. In our institution, CT is routinely obtained as part of the emergency department diagnostic workup for abdominal/pelvic pain of unknown origin to rule out unexpected complications or emergent pathologies. In this case, imaging confirmed the suspected diagnosis, identified the appendix within the hernial sac, excluded perforation or bowel obstruction, and ultimately facilitated operative planning for a minimally invasive approach.

The presence of appendicitis within the hernia sac can complicate standard hernia repair due to the risk of infection, especially when using synthetic mesh [6, 11, 12]. The Losanoff and Basson [6] classification system is often used to guide management, categorizing Amyand’s hernias based on the presence and severity of appendiceal inflammation and systemic infection. In this patient’s case—an edematous appendix without abscess or perforation—the classification would be Type 1, which typically warrants appendectomy and hernia repair with mesh placement [2, 3]. Had the appendix been discovered to be inflamed or perforated, the use of mesh would not have been appropriate given the risk of infection [13].

The decision to perform an appendectomy in noninflamed Amyand’s hernias remains debated. The argument against removal mainly revolves around the idea of maintaining a clean surgical field, whereas violating the integrity of the colonic tract creates a contaminated field that dramatically increases the risk of infection with mesh placement [6, 9]. Some advocate for prophylactic removal to prevent future appendicitis, while others caution against unnecessary procedures, particularly in pediatric populations [14, 15]. Nonetheless, there remains no evidence in the literature that an appendix in the sac has a risk for appendicitis above the average. Ultimately, the decision remains at the discretion of the operating surgeon.

Our patient’s history of chronic constipation may have contributed to increased intra‐abdominal pressure, predisposing to both the initial hernia development and subsequent complications [3, 7]. Hernia patients tend to have significantly higher obstructive defecation and colonic inertia subscale scores, *p*  < 0.01, and constipation severity correlated with hernia type per Nyhus classification with a dose–response relationship [16]. The Wexner constipation scale is scored significantly higher in hernia patient populations and factors as an independent predictor of hernia formation in multivariate analysis [17]. The odds ratio of constipation as an independent risk factor for herniation of any kind is 2.5 [18]. According to the NEJM review on hernias, high intra‐abdominal pressure is associated with increased hernia risk, and ACOEM guidelines also list constipation as an independent factor for herniation [19]. Any sort of abdominal straining during urination or defecation is significantly associated with hernia, in particular with direct rather than indirect hernias [20]. The incidental umbilical hernia further supports this theory. The role of colonoscopy in patients with chronic constipation and new or changing hernia symptoms, particularly beyond age 40, remains an important consideration for broader colorectal evaluation [21, 22].

We chose a robotic transabdominal preperitoneal approach because the patient was hemodynamically stable without perforation or generalized peritonitis, allowing for a minimally invasive repair. The robotic platform was immediately available at our institution and provided excellent visualization for the reduction of the incarcerated appendix, appendectomy, and bilateral preperitoneal mesh repair through the same access. Conventional laparoscopy and open repair were both considered acceptable alternatives; however, given the surgeon’s experience and institutional resources, the robotic approach was favored. The contralateral inguinal hernia was identified intraoperatively within the same preperitoneal dissection. Repairing both defects during a single operation avoided the need for future operation and anesthesia exposure. Because there was no perforation, abscess, or gross contamination, the risk of mesh infection was considered low, and bilateral mesh repair was felt to be appropriate. Informed consent was obtained from the patient for the deidentified analysis, proliferation, and publication of this case.

## 4. Conclusion

Amyand’s hernia is an uncommon clinical entity requiring a high index of suspicion and careful radiologic evaluation. This case illustrates the role of CT in diagnosis and highlights the importance of individualized management strategies based on hernia contents and appendiceal status. Clinicians should consider underlying risk factors such as chronic constipation and delayed colorectal screening when managing similar presentations. Most reported Amyand’s hernias have historically been managed through an open inguinal approach, although minimally invasive laparoscopic and robotic techniques are increasingly described in selected stable patients. Similar to prior reports, our patient experienced an uncomplicated postoperative recovery without mesh infection or recurrent hernia. This case further supports that, in carefully selected patients without gross contamination, a robotic minimally invasive approach can safely facilitate appendectomy and definitive mesh hernia repair while providing the advantages of enhanced visualization and expedited recovery.

## Funding

This research received no specific grant from any funding agency in the public, commercial, or not‐for‐profit sectors.

## Disclosure

After using the tool, the authors reviewed and edited the content as needed and take full responsibility for the content of the publication.

## Consent

Written informed consent was obtained from the patient for the publication of this case report and any accompanying images.

## Conflicts of Interest

The authors declare no conflicts of interest.

## Data Availability

The data that support the findings of this study are available from the corresponding author upon reasonable request.
